# Recommendations for early diagnosis of Developmental Dysplasia of the Hip (DDH): working group intersociety consensus document

**DOI:** 10.1186/s13052-020-00908-2

**Published:** 2020-10-09

**Authors:** Rino Agostiniani, Giuseppe Atti, Salvatore Bonforte, Carolina Casini, Marco Cirillo, Maurizio De Pellegrin, Daniela Di Bello, Francesco Esposito, Ambra Galla, Giorgio Marrè Brunenghi, Nicola Romeo, Paolo Tomà, Norberto Vezzali

**Affiliations:** 1 Department of Pediatrics and Neonatology, ASL Toscana Centro, Florence, Italy; 2 “Marino Ortolani” Center for the early diagnosis and treatment of Developmental Dysplasia of the Hip, Sant’ Anna Hospital, Ferrara, Italy; 3 Family pediatrician, Catania, Italy; 4 Pediatrics Unit, Sant’ Andrea Hospital, Rome, Italy; 5 Imaging Department, Pediatric Hospital Bambino Gesù, Rome, Italy; 6 Childhood Orthopedics and Traumatology Unit, San Raffaele Hospital, Milan, Italy; 7 Orthopedics and Traumatology Unit, Burlo Garofalo Hospital, Trieste, Italy; 8 Emergency Imaging Diagnostic Unit, A.O.R.N. “Santobono-Pausilipon”, Naples, Italy; 9 Orthopedics and Traumatology Unit, Giannina Gaslini Institute, Genoa, Italy; 10 Pediatrics Unit, State Hospital, San Marino, Republic of San Marino; 11 Radiology Services, Bolzano Health District, Bolzano, Italy

**Keywords:** Developmental dysplasia of the hip, Intersociety consensus, Early diagnosis, Hip osteoarthritis

## Abstract

This consensus document has been prepared by a multidisciplinary group of experts (Paediatricians, Radiologists, Paediatric Orthopaedics) and it is mainly aimed at paediatricians, hospitals and primary care providers. We provide recommendations for the early diagnosis and treatment of Developmental Dysplasia of the Hip (DDH) and indications on its management.

## Introduction

Developmental Dysplasia of the Hip (DDH) is the most common congenital disease of the musculoskeletal system in newborns. The disease ranges from a simple flattening of the acetabular cavity to the complete dislocation of the femoral head. If untreated, DDH can cause early hip osteoarthritis and, in the most severe forms, the presence of a limp with severe functional limitations, since walking age.

An early diagnosis, which is essential for an early treatment, is the fundamental prerequisite in order to achieve the best treatment results and to reduce the possibility of hip osteoarthritis in young adults. The treatment effectiveness is maximized when it begins early, within the first month or, if possible, the first days of life.

Paediatricians, radiologists, paediatric orthopedics worked together to design and write this document. The aim is to provide recommendations for early diagnosis and treatment of hip developmental dysplasia (DDH) and indications on its management.

## Methods

### Methodology used

The document has been prepared according to the following steps:

1. identification of a multidisciplinary group of experts (Paediatricians, Radiologists, Paediatric Orthopaedics) with all the skills required to draft the document;
2. formulation, by the group of experts, of the most relevant scientific questions, with particular attention to the areas of major interest (opportunities for DDH screening, diagnostic tests, method of execution and timing of the tests, selective or universal ultrasound screening, operators’ training, need for data registration);
3. review of the scientific literature according to a research strategy capable of identifying the best available scientific evidence relating to the questions raised;
4. evaluation and synthesis of the bibliography collected;
5. discussion and approval of the results’ analysis with a *consensus conference* and formulation of recommendations based on the experts’ advice;
6. drafting of the final shared document;
7. presentation and publication of the final version of the *consensus*
8. organization of a review in 3 years.

### Review of scientific literature

From 1st of January 2000 to 31st of March 2019 a bibliographic research was performed by consulting Medline’s databases through PubMed. Studies in English and Italian and articles in other languages have been included only if particularly significant. The members of the working group have identified keywords used for the research strategy. Articles taken from bibliographic references of the initially selected studies have also been considered. The references were periodically updated during the *consensus* drafting. Abstracts and articles were then evaluated by the working group, who selected the relevant articles favouring, where present, meta-analyses of clinical trials, systematic reviews, randomized controlled clinical trials, cohort studies and general interest articles. Additionally, a specific search was performed in order to identify guidelines and other national and international consensus documents already available. The articles considered most relevant according to the AGREE II methodology (1) were shared among all panel members and discussed in regular dedicated frontal meetings. A final review of the literature was performed before the final draft.

## Methodological references

1. Brouwers MC, Kerkvliet K, Spithoff K. The AGREE Reporting Checklist: a tool to improve reporting of clinical practice guidelines. BMJ 2016;352:i1152

## Consensus

### Disease

DDH is the most common congenital disease of the musculoskeletal system in newborns. The disease ranges from a simple flattening of the acetabular cavity to the complete dislocation of the femoral head [1]. If untreated, DDH can cause early hip osteoarthritis [2, 3] and, in the most severe forms, the presence of a limp with severe functional limitations, since walking age [4].

### DDH epidemiology

The analysis of the literature does not allow a precise definition of DDH epidemiology due to the following reasons:

- the classification and definition of the disease have changed over time, in relation to the diagnostic methods used;
- the tools available for the diagnosis are characterized by a different accuracy (radiographic, clinical, ultrasound examination);
- DDH prevalence changes according to the children’s age at the time of the study and their ethnicity.

In the past, the incidence of severe forms of the disease (complete dislocation of the femoral head), without an early diagnosis program, was reported to be 0.13% of all newborns [5]. The actual DDH frequency undoubtedly exceeds this value, since the disease includes not only complete dislocations but less severe clinical pictures, characterized by dysplasia of the acetabulum with the femoral head still in place, which are potentially responsible for early osteoarthritis of the hip; these features, detectable by ultrasound examination, are present in 1.6% of the general population [6].

### The natural history of DDH

The femoral head must be positioned in a stable and entirely congruent manner within the acetabular cavity to ensure the healthy development of the child’s hip. Children with a complete dislocation of the femoral head, if untreated, will maintain a dislocated hip and present a limp at the beginning of gait. The spontaneous evolution of the less severe forms of DDH has not yet been fully defined because of the objective ethical difficulty of performing a methodologically correct randomised clinical trials involving the comparison between the “treated” and the “untreated” groups. However, retrospective observational studies available in literature, report that DDH, if not detected and treated promptly, entails an increased risk of corrective surgery and hip replacement surgery [3, 7].

The clinical signs detectable in the first months of life may disappear overtime, differently from the pathological morphological patterns at the acetabulum level. According to Furnes [8], failure to treat these cases contributes to 29% of arthroplasty performed under the age of 60.

### Importance of early diagnosis of DDH

An early diagnosis, which is essential for an early treatment, is the fundamental prerequisite in order to achieve the best treatment results and to reduce the possibility of hip osteoarthritis in young adults [9–15]. The treatment effectiveness is maximized when it begins early, within the first month or, if possible, the first days of life [16, 17]. If a hip dislocation is present at birth, the anatomical alterations secondary to the dislocation of the femoral head are not yet consolidated; instead, they may be consolidated in delayed treatment, after the second or third month of life of the child. In the latter case, the reduction of the femoral head within the acetabular cavity is much more problematic and sometimes impossible with a closed approach. Furthermore, any minimal residual alteration of the acetabulum, most likely in late treatments, may lead to hip osteoarthritis in adulthood [9].

There are studies which analysed the time limit within which the potential for acetabular growth, and therefore for the healing of dysplasia, is still high. The ideal time limit for diagnosis and treatment has been identified as the sixth week of life [18]; beyond that age, a complete normalization of the acetabulum after treatment is not guaranteed.

An essential role regarding the incidence of avascular necrosis of the femoral head, the most feared complication in DDH treatment, is determined by early diagnosis. Bradley, in patients treated with closed reduction and cast, reports an average incidence of avascular necrosis of 10%, with a progressively higher frequency of cases in relation to the age at which treatment is started [19]. Also Senaran [20] highlights that treatment is more complex and lasts longer in cases of late diagnosis.

### DDH diagnosis

The examinations available for the diagnosis of the DDH are *clinical, ultrasound and radiographic examinations.*

#### Clinical examination

Clinical examination of the hips, at birth and in the first month of life, continues to play a fundamental role in the diagnosis of DDH, particularly in the severe forms of the disease [21].

The correct clinical examination of the hips requires the evaluation of: i) the child’s spontaneous posture; ii) a possible leg length discrepancy, in particular of the thighs (Galeazzi sign); iii) an asymmetry of the lateral profile of the pelvis; iv) a decreased hip range of motion during abduction of the thighs (not a very specific finding, as it is also present in healthy children who have maintained for a long time intrauterine postures with the lower limbs in adduction) [6]. Only after the above evaluations, will the examiner perform the Ortolani manoeuvre [22, 23] followed by the Barlow manoeuvre [24]. When the femoral head is completely and permanently located outside the acetabulum, anatomical conditions can prevent reduction with the simple Ortolani manouvre [25]; in these cases, we will not detect the “clunk”, but only a severe limitation of the thighs abduction. Articular noises such as “clicks” or “creaks” should not be considered pathological findings.

#### Ultrasound examination

The introduction of the ultrasound technique for the study of childhood hip diseases is undoubtedly the most important novelty for the diagnosis of DDH in the past 30 years. Ultrasound examination allows to visualize with precision all the components, mineralized and not, of the infantile hip and to recognize any alteration of the hip joint from the first days of life.

The main techniques proposed for the ultrasound study of the hip are:

1. **Graf technique** [26, 27]: initially used in German-speaking countries and in Italy, but now widespread throughout the world. This method of study is of rapid execution, well standardized and allows to identify with precision and in detail the morphology of all the joint components. By angle measurements of bony and cartilaginous components of the acetabulum, it allows the classification of normal and pathological images according to progressive severity criteria.
2. **Harcke technique** [28]: it evaluates the femoral head stability at rest and under stress (method used mainly in the USA), by using different scans, longitudinal and coronal, and by extending or flexing the thigh at 90° with respect to the pelvis,
3. **Morin-Terjesen technique** [29, 30]: based on the calculation of the percentage of bone coverage of the femoral head (mainly used in Scandinavian countries).

Regardless of the technique used, great attention should be paid to the training of sonographers and the periodic verification of their skills.

All methods must be performed in strict compliance with the author’s instructions. Diagnostic errors can be avoided by strictly adhering to the technique used; the accuracy of the examination, especially regarding specificity, is closely linked to the operator’s technical skills. Only adequately trained and certified operators should perform the ultrasound examination in newborns [31, 32]; from a medical-legal point of view, according to the regulations in force in our Country, the hip ultrasound is to be considered a “medical act”, thus constituting an assumption of professional responsibility (in terms of expertise, prudence and diligence) by the operator.

#### Radiological examination

Hip X-ray still plays a role in the diagnosis of DDH [33]. However, the method is useful only from the 3rd – 4th month of life of the child, when the skeletal structures reach a sufficient degree of mineralization and may be visualized by X-rays. The risks associated with radio exposure and the modest information that the examination provides in the first 3–4 months of life, have made this diagnostic tool no longer recommended as a screening test for DDH. Hip X-rays must be used as a second-level diagnostic investigation in order to: i) confirm a clinical or ultrasound suspicion of DDH; ii) as a follow-up the disease; iii) document the complete recovery of the most severe forms; iv) highlight the possible onset of complications.

### Comparison between the results of the clinical and the ultrasound examinations

Studies comparing the results of clinical and ultrasound examinations have shown that the latter is more sensitive to detect all children with DDH [34, 35]. The concordance between clinical and ultrasound examinations is suitable in severe DDH cases (type III and IV according to Graf), but unsatisfactory for less severe cases (type IIc, D, IIb according to Graf) [35].

### DDH screening

The need for DDH screening, aimed at an early diagnosis is now widely shared [21]; the following points are still being debated:

- diagnostic tests to be used and data recording methods;
- necessary screening execution time;
- opportunity to follow a “universal” programme (aimed at all newborns) or a “selective” programme (for children with risk factors).

Historically, X-ray screening of the hip at the age of 4–6 months has been recommended and implemented on the basis of the Health Authorities recommendations, especially in DDH endemic regions, such as Brianza and Emilia-Romagna. The need to reduce radio-exposure, while maintaining a high level of attention to this disease, suggested to consider as a significant progress the identification of any clinical sign that raised its suspicion [22]. For many years, the screening for DDH “early” diagnosis has been performed in many parts of the world using the Ortolani manoeuvre [23], later associated to Barlow’s [24].

Many publications over the years have highlighted the limitations of the clinical screening [34–37]. If it is true that in the presence of a positive sign of Ortolani the ultrasound examination always documents the presence of DDH, it is also true that the absence of such sign is not a guarantee of its absence. It should also be emphasized that a negative clinical sign does not always represent the normalization of an unstable hip, but sometimes a worsening of its morphological aspect, with an irreducibility of the dislocation [25].

Indirect data that clinical screening has not allowed an early detection of all forms of DDH and the implementation of effective treatments are extractable from:

- Regional Register of Orthopaedic Prosthetic Implantology of the Emilia-Romagna Region (RIPO) [2]: DDH represents the second cause of arthroplasty, with an incidence of 10.9% between 2000 and 2011; the incidence rises up to 31.1% in patients operated on under 40 years of age;
- The Norwegian Arthroplasty Register [3]: between 1987 and 2007, 163 of the 713 patients (22,86%) treated under 40 years of age with hip arthroplasty presented a DDH; out of these young adults 82% were females and 18% males and the average age at diagnosis was 4.4 years in the first and 22 years in the second.

These data suggest that, although the Scandinavian countries and the Emilia-Romagna Region have always been aware of the importance of the disease and are in favour of carrying out a clinical examination as a tool for DDH screening, the problem of missed or late diagnosis has not been completely solved and the disease still represents a significant health concern.

The ultrasound examination of the hips, introduced by the end of the 1980s, represented a major technological advance, initially as a useful tool for a more accurate diagnosis, then assuming the possible role of a screening test of DDH.

In the first 10 years of 2000, most of the papers published on DDH ultrasound screening provided indications for a “selective” programme (clinical examination for all newborns and ultrasound examination only for patients with clinical or anamnestic risk factors) [38, 39].

Data from more recent studies, in the last 10 years show that programs involving “selective” ultrasound screening have not significantly changed the number of late diagnoses of the disease and the number of children who undergo surgery. In patients without risk factors that escape selective screening, DDH diagnosis is late, thus leading to a higher incidence of complications. Interesting is the data of a review carried out by Sink [7]: he examined patients who underwent surgery as the consequence of a late treatment of DDH: 85.3% of these did not meet the inclusion criteria for the selective screening, i.e. they did not present any risk factor. Broadhurst [40] reports that in the United Kingdom there has not been a decrease in DDH late diagnoses since the introduction of selective ultrasound screening; in both authors’ interpretation this is explained by the fact that many of the children with a late diagnosis had no risk factors at birth and escaped the selective ultrasound screening. The results of these studies indicate that selective ultrasound screening, aimed only at patients with risk factors, is doomed to fail due to the inability to identify those children affected by DDH, but without clinical and/or anamnestic risk factors. Conversely, the results obtained in countries that have introduced a “universal” ultrasound screening programme show a significant reduction in late diagnosis and number of children undergoing interventional treatment. Biedermann [41] reports the results of a universal ultrasound screening conducted in Austria showing how early diagnosis has significantly reduced both open surgeries, to reduce the femoral head within the acetabular cavity (0.04 per thousand live births), and closed reduction interventions (0.86 per thousand live births). Moreover, similar results had already been reported by previous authors: Von Kries [42] indicated a reduction in surgery of 52%, while Thallinger [43] a reduction of 46% (from 1.3 per thousand to 0.7 per thousand of live births); Thaler [44] in a comparison between two screening periods, namely 1978–1982 (clinical screening) and 1993–1997 (universal ultrasound screening) reported a reduction in surgery of 85%. In some Countries the application, of the ultrasound method as a universal screening tool for newborns has raised the problem of the higher percentage of “ultrasound” diagnosis of dysplasia compared to “clinical” diagnosis [45, 46]. Actually, if this doubt could arise in the first periods of application of the method, the data reported in subsequent years have reduced the problem, in particular, the fear of a possible “higher incidence of treated hips” (overtreatment).

The technological progress of the equipment, together with the improvement of the knowledge resulting from the screening experience, has allowed refining the evaluation of DDH. In countries where the universal ultrasound screening has been implemented, the percentage of children treated for DDH decreased compared to the previous period when screening was carried out only by clinical examination. Thaler [44] also reports a 48% reduction in abduction treatment, while Thallinger [43] indicates a 2.6% reduction in treated children. These percentages are in line with the 2.6% percentage reported in Central Europe [34] and lower than that reported by the Norwegian register based on radiographic diagnosis, which is 3.3.%.

Regarding the economic costs associated with the different DDH screening programmes, it has been demonstrated that universal ultrasound screening ensures a significant reduction in the health costs associated with the most invasive surgical treatments; by adding these costs to those necessary to organise set up a universal ultrasound screening programme, the overall economic costs of the different screening programmes are sustainable [44, 47, 48].

### Data collection

Regardless of the technique used, any reliable DDH screening programme must include the systematic and computerised collection of data on the results of the health interventions implemented.

The creation of a regional computerized registry for the collection of screening data and admissions with DRG related to DDH therapy is an essential tool to verify the results of the programme to prevent disease outcomes.

Only through data analysis it will it be possible to assess the effectiveness of the programme, both in terms of health protection and costs, for the individual and the community.

## Conclusions

- ➢ All newborns must undergo a clinical examination of their hips by a neonatologist or paediatrician at birth; the examination must be properly recorded.
- ➢ The clinical examination of the hips must be repeated during health assessments in the first 6 months of life by the family paediatrician, and properly recorded.
- ➢ All newborns who present a “clunk sign” at clinical examination must undergo an ultrasound examination of their hips before being discharged from the birth point or, in any case, within the first week of life; the examination must be properly recorded. It is crucial to organize a regional DDH screening program, including all newborns, with hip sonography at 4–6 weeks of life, and a registry for screening results and DDH treatments.
- ➢ All newborns, regardless of the presence of risk factors, must be included in a program
  of DDH screening that foresees the performance of an ultrasound examination of the hips between 4 and 6 weeks of life by certified operators and the creation of a computerized regional registry for
  the collection of screening data and DRG admissions related to DDH treatment.
- ➢ Health services should identify a local screening and care pathway, shared by paediatrician, orthopaedic and radiologist, for all cases with a positive dysplasia ultrasound examination (type IIb, IIc, D, III, IV image according to Graf’s classification); type IIa hips should be monitored with ultrasound examination and treated only in the absence of signs of adequate maturation.
- ➢ Health services, with the collaboration of scientific societies, must: i) identify the centres suitable for carrying out the screening; ii) implement specific training programs for learning the clinical and ultrasound examination of the hips; iii) provide certification methods for the operators dedicated to the ultrasound examination; iv) verify the quality of the services provided.

## Data Availability

Not applicable.

## Abbreviation

DDH: Developmental Dysplasia of the Hip
